# Preparation, physicochemical characterization and antimicrobial activities of novel two phenolic chitosan Schiff base derivatives

**DOI:** 10.1038/s41598-018-29650-w

**Published:** 2018-07-30

**Authors:** Mohamed A. Hassan, Ahmed M. Omer, Eman Abbas, Walid M. A. Baset, Tamer M. Tamer

**Affiliations:** 1 Protein Research Department, Genetic Engineering and Biotechnology Research Institute (GEBRI), City of Scientific Research and Technological Applications (SRTA-City), New Borg El-Arab City, P.O. Box, 21934 Alexandria, Egypt; 2 Polymer Materials Research Department, Advanced Technologies, and New Materials Research Institute (ATNMRI), City of Scientific Research and Technological Applications (SRTA-City), New Borg El-Arab City, P.O. Box, 21934 Alexandria, Egypt; 3 Zoology Department, Faculty of Science, Alexandria University, Alexandria, Egypt; 4 National Organization for drug control and Research (NODCAR), 51 Wezaret El-Zeraa st., Dokki, Cairo, Egypt

## Abstract

This study intends to develop novel two antimicrobial phenolic chitosan Schiff bases (I) and (II) via coupling of chitosan with Indole-3-carboxaldehyde and 4-dimethylaminobenzaldehyde, respectively, for boosting the antimicrobial activity of native chitosan. The alterations in the chemical structure and morphology of the Schiff bases were verified using FT-IR, electronic spectrum analysis, and SEM, whereas the thermal properties were investigated by TGA and DSC instruments. The results obtained from the potentiometric analysis referred that the degrees of substitution were 1.15 and 12.05% for Schiff bases (I) and (II), respectively. The antimicrobial activities of Schiff base (I) were significantly augmented more than Schiff base (II) and chitosan. Minimum inhibitory concentration (MIC) of Schiff base (I) was perceived at 50 µg/ml against tested microorganisms except for *B*. *cereus* and *C*. *albicans*. The highest concentration of Schiff base (I) could inhibit the growth of Gram-positive up to 99%. However, Schiff base (II) recorded the maximum inhibition rate versus Gram-positive approximately 82%. The cytotoxicity of the developed materials was estimated by MTT assay that substantiated their safety to fibroblast cells. The findings emphasized that the developed Schiff bases might be implemented as antimicrobial contenders to pure chitosan for treatments of wound infections.

## Introduction

Antimicrobial bio-polymers have been extensively studied for the last years, which considered the key to the vast majority of several applications such as wound dressing^1^, tissue engineering^2^, medical textile^3^, packaging^4^ and water treatment^5^. Among all natural bio-polymers, chitosan is easily available, found in several insects and microorganisms and considered one of the most effective antibacterial bio-polymers^6,7^. Chitosan can be easily extracted from chitin by simple deacetylation process and composed from randomly distributed deacetylated unit (β-(1 → 4)-linked d-glucosamine) and acetylated unit (N-acetyl-d-glucosamine) copolymer^8^. The presence of deacetyl amine groups along polymer backbone provided its basic character and simplified its solubility in the acidic medium. The unique properties of chitosan such as non-toxicity, anti-bacterial activity, biodegradability and excellent biocompatibility^9,10^ renders it widely used in the bio-medical applications as a drug carrier^11^, antimicrobial^12^, antioxidant^13^, antitumor^14^, and a wound dressing agent^15,16^. Therefore, chemical modifications of chitosan simplify its chemical transformation through the present NH_2_ and OH^−^ groups and allow the formation of several functional derivatives such as sulfonation^17^, amination^18^ and carboxymethylation^19^. In addition, chitosan can be chemically modified for widen its applications via grafting with functionalized monomers^20,21^ and formation of polyelectrolyte complexes with other anionic polymers^22^. As it well known that chitosan has a decent antimicrobial activity against several types of bacteria, and these activities are interrelated to the quantity of the adsorbed chitosan on the cells of bacteria^23,24^. The activity of the prime chitosan against microbial infections has become increasingly inhibited due to the incessant mutations of microorganisms coupled with reduced rates of establishment of new antimicrobial agents. Accordingly, scientists are struggling to synthesize novel antimicrobial chitosan derivatives to obstruct the wound infections. Several studies have reported the significant effect of antimicrobial biopolymers and chitosan itself to enhance the wound healing process through preventing the wound infections that result in tissue maceration^23,25–27^. It has been reported that chitosan modified diisocyanate (DIMC)^28^, O-quaternary ammonium N-acyl thiourea chitosan^29^, chitosan-thioglycolic acid^30^, kanamycin-chitosan nanoparticles^31^, crosslinked chitosan^32^ and O-amine functionalized chitosan^33^ exhibited better antibacterial activity degrees against *Escherichia coli*, *Staphyloccocus aureus* and other microorganisms. Likewise, chitosan Schiff base derivatives are considered one of the best choices for increasing antimicrobial activity of chitosan, since carbonyl groups of aldehyde or ketone can efficiently couple with NH_2_ groups of chitosan to form the corresponding chitosan Schiff base with imine characteristic group (-RC=N-)^34^. This leads directly to altering chitosan molecular structure, enhancement its hydrophilicity as well as increasing the positively charged ions, which results in better antibacterial activity compared to the unmodified chitosan. Numerous chitosan Schiff bases such as chitosan derivative-para-substituted benzaldehydes, chitosan-crotanaldehyde and chitosan-4- chlorobenzaldebyde Schiff bases have been developed and examined their antibacterial and other biological activities, etc.^35–37^. The present work addresses the development of antimicrobial chitosan derivatives for wound dressing applications that could expedite the wound healing through hindering the microbial wound infections. Herein, two new phenolic chitosan Schiff bases were synthesized via coupling of chitosan with Indole-3-carboxaldehyde and 4-dimethylaminobenzaldehyde to form chitosan Schiff bases (I) and (II), respectively. The developed Schiff bases were characterized and verified using different characterization tools. Furthermore, their antimicrobial activities were examined against various pathogenic microorganisms that frequently provoking wound infections as well as, their cytotoxicity studies were also evaluated.

## Results and Discussion

### Determination of free amines via potentiometeric method

**Figure 1:**
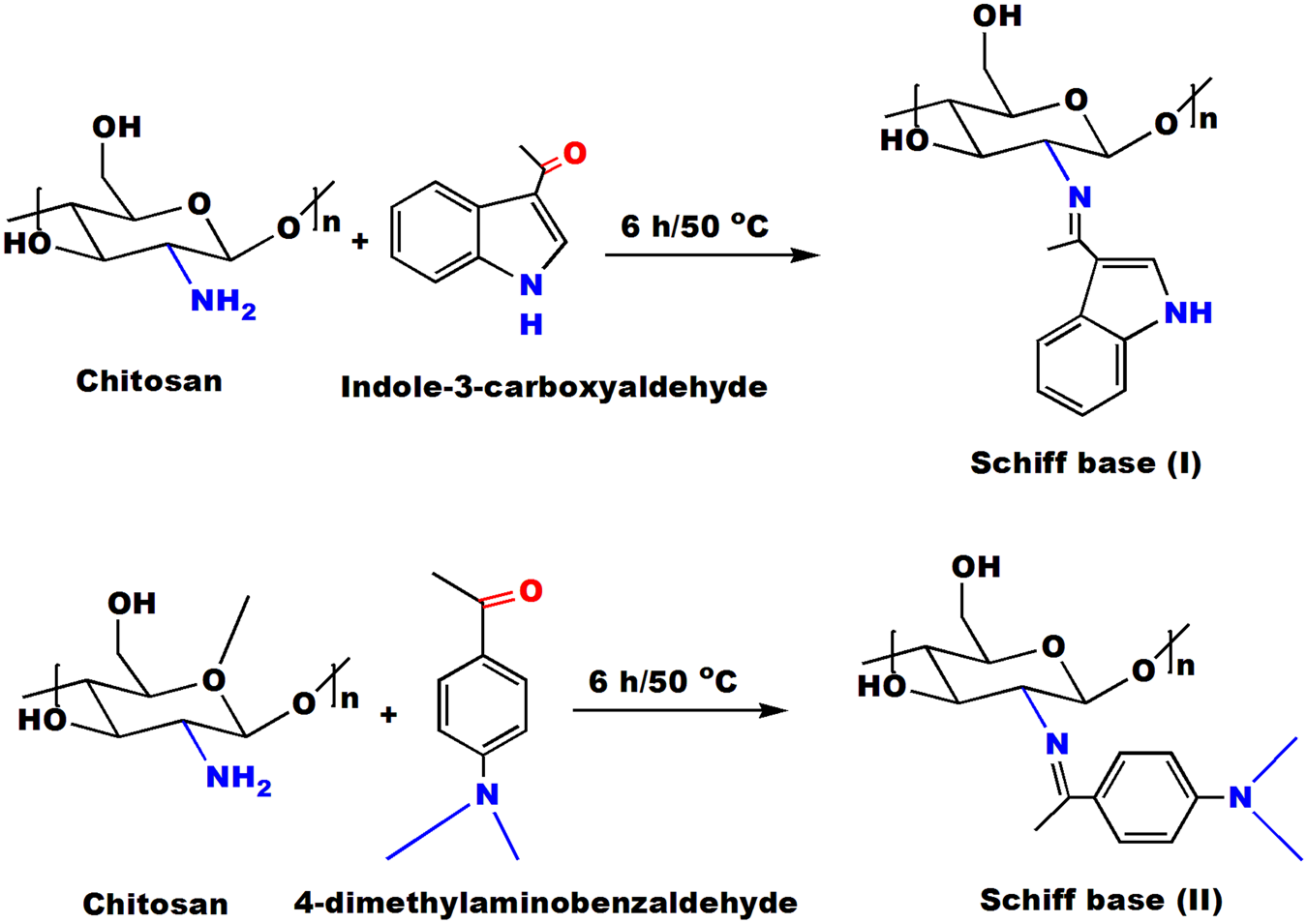
Synthesis scheme of chitosan Schiff bases (I & II).

**Figure 2:**
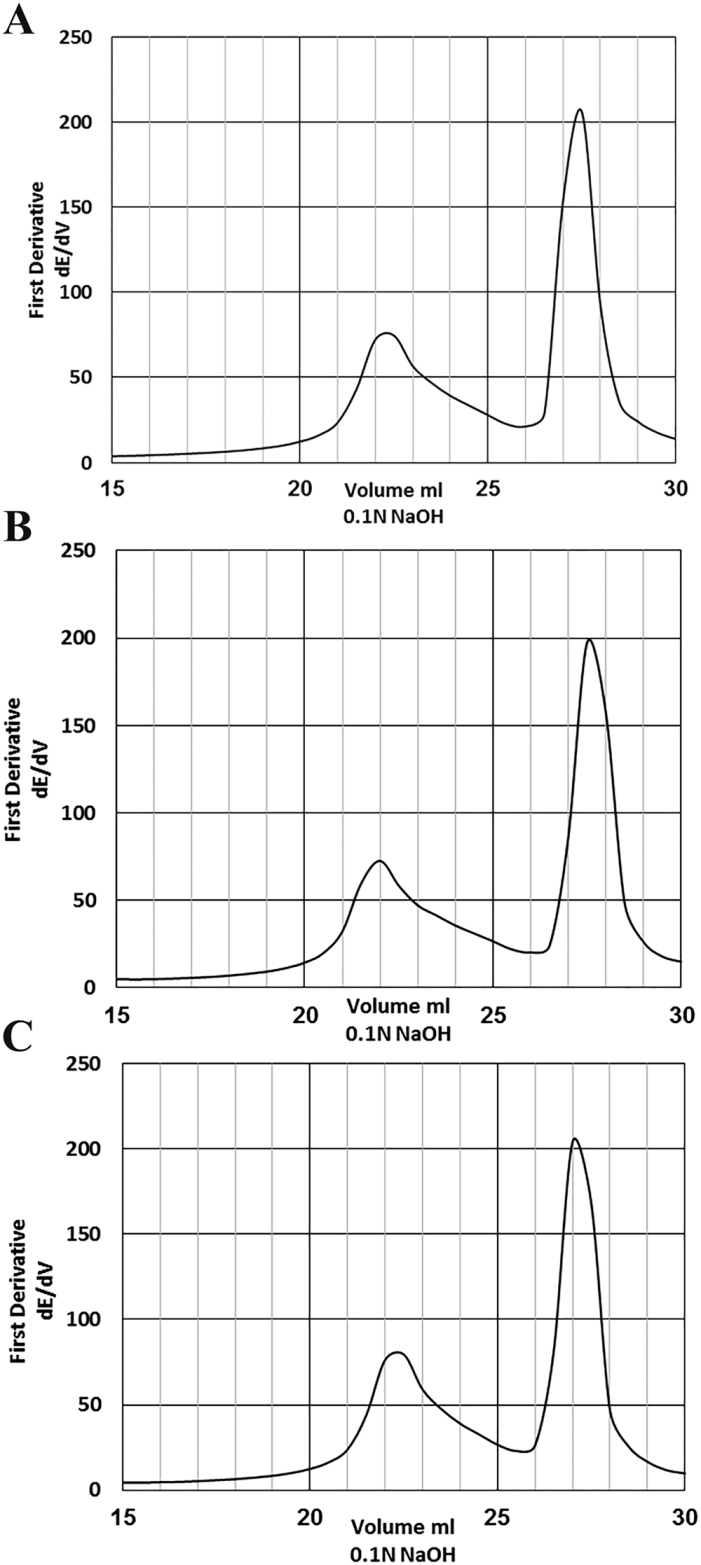
Potentiometric titration of **(A)** chitosan, **(B)** Schiff base (I) and **(C)** Schiff base (II).

Figure 1 represents a proposed mechanism for the synthesis of chitosan Schiff base derivatives. Actually, there are several common methods have been adopted for determination the degree of deacetylation (DD) of chitosan via measuring its free amine groups. Since, it’s considered one of the most essential parameters, which directly affect the properties and applications of chitosan. These methods have been conducted by using FT-IR^38^, NMR^39^, UV-spectrophotometric analysis^40^, colloidal titration^41^, and potentiometric titration^42^. The latter approach was applied in the current study, where chitosan-HCl solution was titrated with an alkaline NaOH solution. Figure 2 exhibits the potentiometric titration for the prepared Schiff bases, and the obtained titration curve has been expressed by two inflection steps. These two steps associated with the equivalence two titration points for the excessive HCl and protonated chitosan, respectively. The degree of substitution could be estimated via determination the content of free amine groups in chitosan before and after reaction.

Table 1 represents the calculated values of DD for chitosan and its two Schiff base derivatives. Moreover, the degree of substitution (DS) was 1.15% for Schiff base (I) and 12.05% for Schiff base (II).

**Table 1:** DD and DS values obtained from potentiometric titration method for chitosan and its Schiff base derivatives.

Sample	DD (%)	DS (%)
Chitosan	93.15	—
Schiff base I	92.0	1.15
Schiff base II	81.1	12.05

### FT-IR analysis

**Figure 3:**
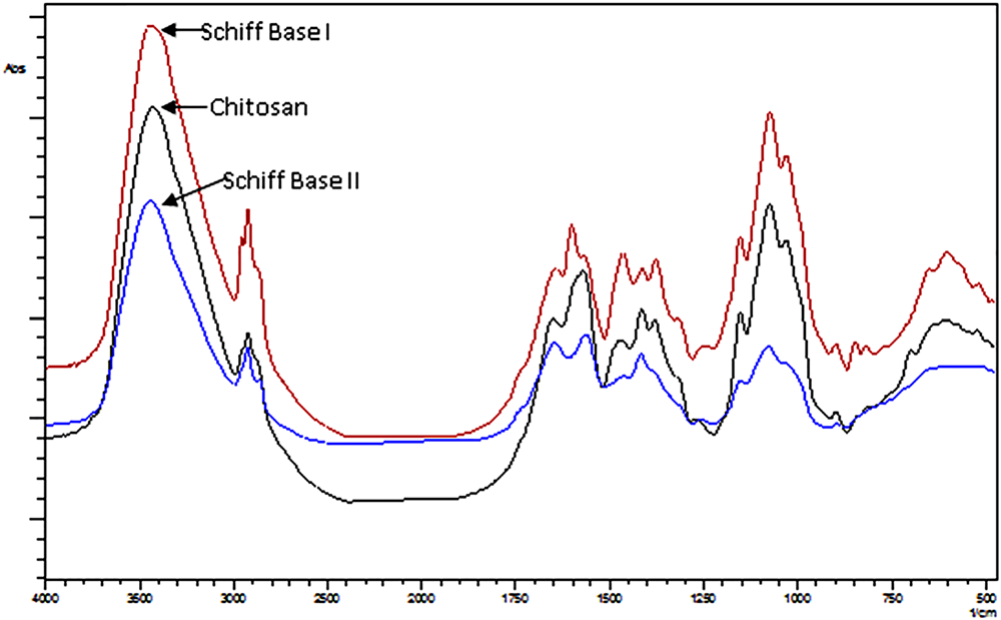
FT-IR spectra of chitosan and Schiff bases (I & II).

Figure 3 demonstrates the FT-IR spectral analysis of neat chitosan and its two Schiff base derivatives (I) and (II). The spectrum illustrates a typical band of polysaccharides such as common bands between 3200–3400 cm^−1^ corresponding to hydroxyl and amine groups. 2960 and 1933 cm^−1^ refer to stretching vibration of CH and CH_2_. 1645 and 1565 cm^−1^ assigned to amide (I). The band at 1370 cm^−1^ corresponding to NH_2_ bend vibration. Where multipacks between 1200–1000 cm^−1^ associated with glycoside C-O, C-O-C and C-C bond^21^.

### Electronic spectra

**Figure 4:**
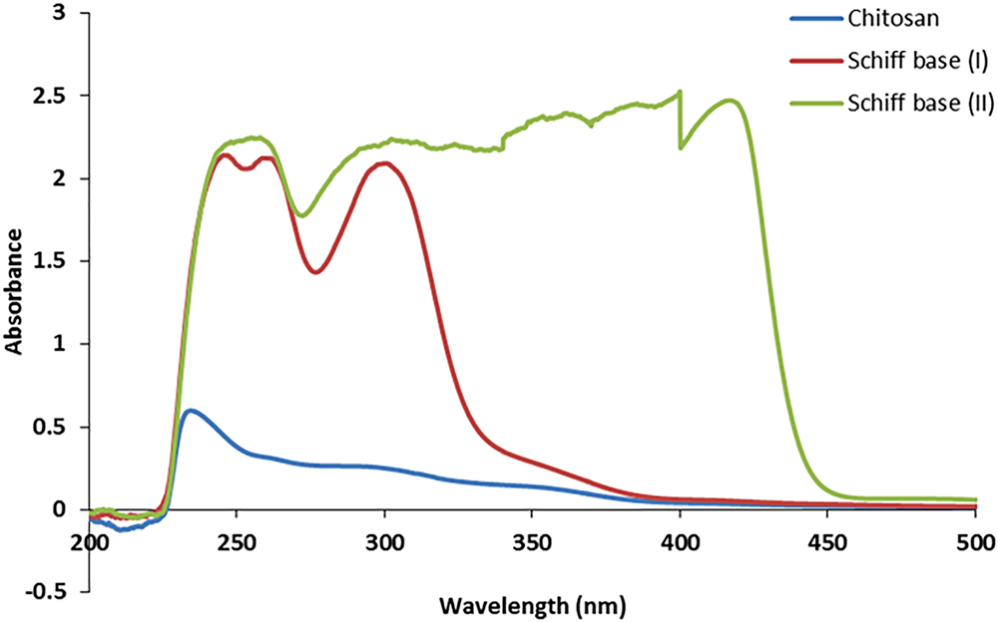
Electronic spectra of chitosan and Schiff bases (I & II).

Figure 4 investigates the UV-visible spectra for chitosan and its new Schiff bases. It was obvious in case of chitosan that the apparent absorbance band at maximum 230 nm could be attributed to n-σ* transition of amine free electrons^43^. Conversely, in case of Schiff bases (I) and (II), the increase in the peak intensity as well as, the observed shift to the higher wavelengths 247 and 361 nm, respectively, could be explained by increasing in donor ability of the substituent to stabilize the excited state^44^. Schiff base bond displays a red-shift, which refers to the attachment of indole-3-carboxaldehyde and dimethylaminobenzaldehyde to amine groups along chitosan backbone^45,46^. Furthermore, generation of new bands at higher wavelength from 259.6 to 300 nm for Schiff base (I) and multibands from 361 to 421 nm for Schiff base (II) could be ascribed to the formation of Schiff base bond –N=C, which creates a new transition n-π* at a higher wavelength.

### Thermal analysis

**Figure 5:**
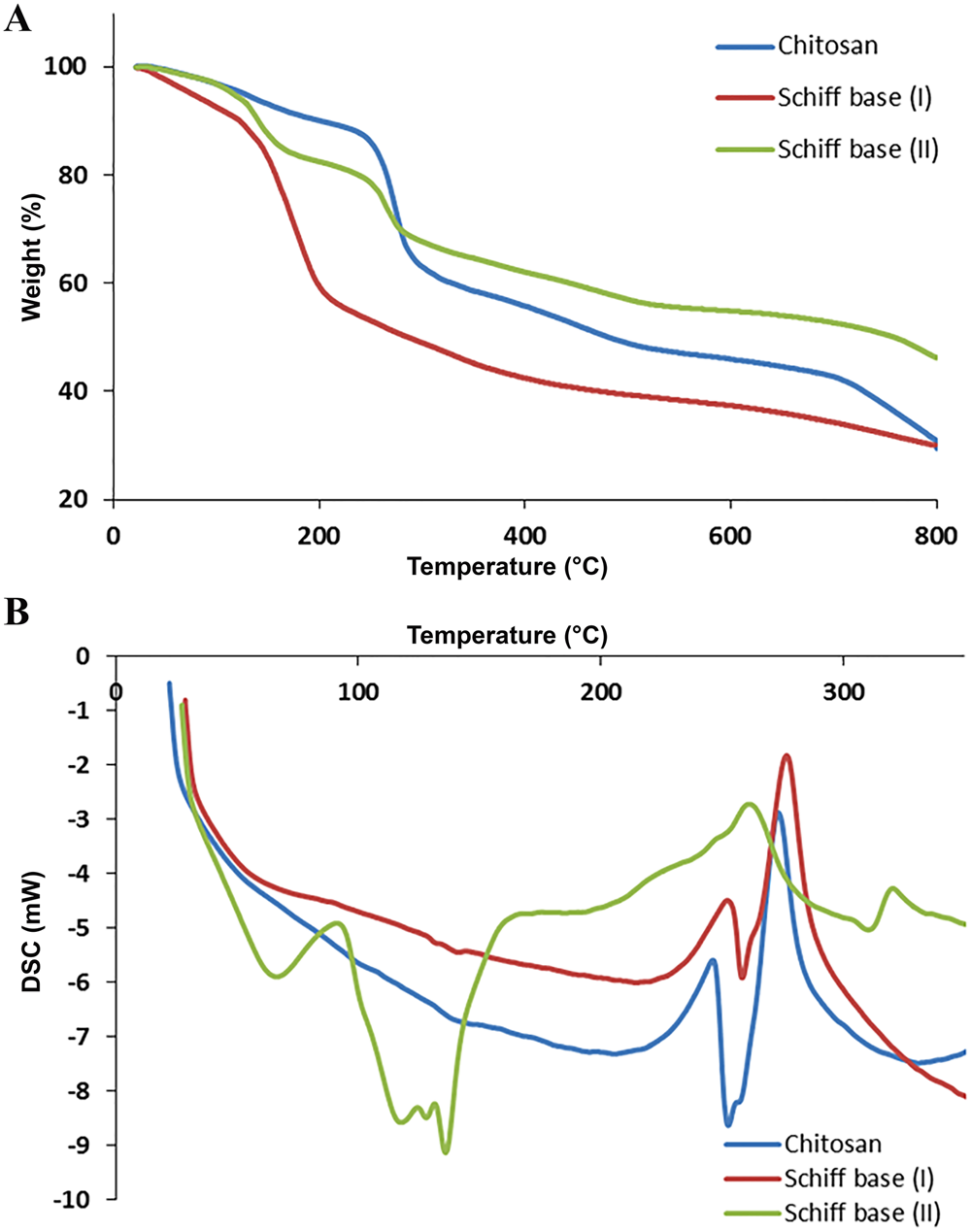
**(A)** TGA and **(B)** DSC of chitosan and Schiff bases (I & II).

Figure 5A exhibits TGA thermograms of chitosan and its two different Schiff bases (I & II). Chitosan demonstrates three regular transition stats. The first stage may due to elevation physically adsorbed moisture up to 120 °C that between 4–5% of polymer weight^47^. Where the second depression of samples weight begin from 230 to 300 °C was attributed to the thermal decomposition of pyranose ring along polymer backbone to form complex adduct^48^. The third decomposition attributed to thermal decomposition obtained from adducts. Prepared chitosan Schiff bases demonstrate thermal behavior varied than chitosan. Table 2 illustrates different degradation steps of Schiff bases compared to chitosan itself. In conclusion, Chitosan shows slightly high thermal stability than Schiff base (I) where it’s less stable than Schiff base (II). On the other hand, DSC is useful tools to characterized thermal properties of chitosan and its derivatives. Figure 5B illustrates DSC thermograms of chitosan and its Schiff base derivatives (I & II) from ambient temperature up to 350 °C. DSC spectrum of chitosan shows a broad endothermic peak around 100 °C that may associate to dehydration of water content. Chitosan and Schiff base (I) exhibit exothermic bands at 247 °C and 250 °C, respectively, that corresponding to pyranose ring thermal decomposition^49^. In the other hand, Schiff base (II) show more complicated exothermic bands that may be attributed to its modification.

**Table 2:** TGA parameters of chitosan and its two Schiff base derivatives.

Sample	1^st^ Depression Ambient- 120 °C	2^nd^ Depression		3^rd^ Depression		4^th^ Depression		T_50_ (°C)
	Weight loss (%)	T (°C)	Weight loss (%)	T (°C)	Weight loss (%)	T (°C)	Weight loss (%)	
Chitosan	4.28	230–300	25.48	300–800	33.57	—	—	481
Schiff base (I)	9.49	140–200	27	200–800	29.2	—	—	287.1
Schiff base (II)	5.39	120–165	9.56	230–280	10.94	518–800	10.02	481.5

### SEM analysis

**Figure 6:**
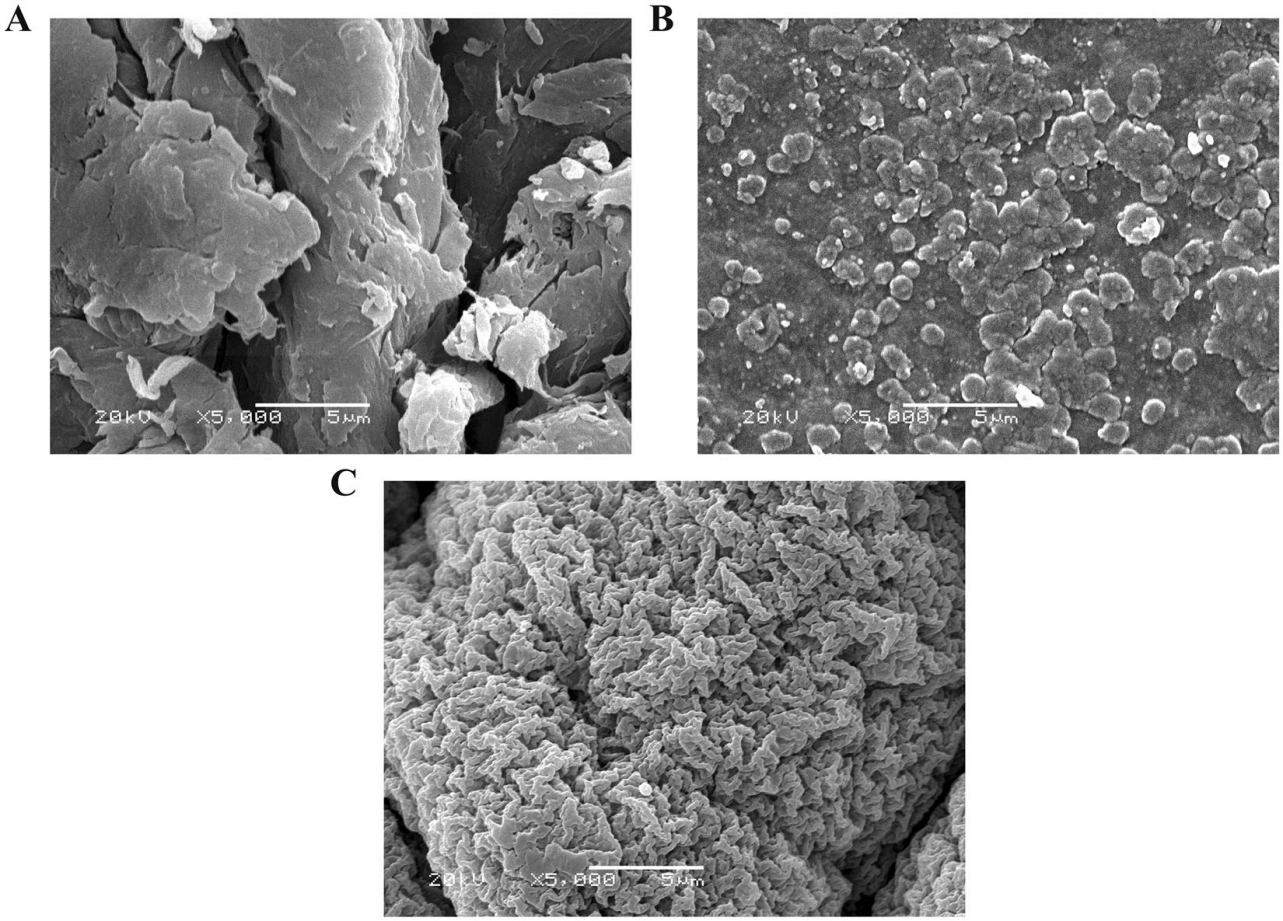
SEM images of **(A)** chitosan, **(B)** Schiff base (I) and **(C)** Schiff base (II).

Figure 6 displays the morphological structure of chitosan, Schiff base (I) and Schiff base (II). The surface roughness and pores of chitosan derivatives were increased than chitosan itself. This behavior was significant in Schiff base (II) (DS = 5.9–12.05) rather than Schiff base (I) (DS = 1.15–1.9). Interruption of the polymeric chain can explain this observation as a result of the coupling of chitosan amine groups with aldehyde.

### Antimicrobial assay using Agar-well diffusion method

Over the recent years, Antimicrobial activities of chitosan and its derivatives have been drawing considerable attention. In addition, mutations of microorganisms to resist the action of antimicrobial materials have enhancing scientists to develop new antimicrobial materials have potential activity against the new version of pathogenic microorganisms. Antimicrobial activities of chitosan and the two developed Schiff bases were initially determined using agar-well diffusion technique against representative Gram-positive bacteria (*S*. *aureus* and *B*. *cereus*) and Gram-negative bacteria (*E*. *coli*, *P*. *aeruginosa*, and *Salmonella* sp.) in addition to yeast strain (*C*. *albicans*). For bacterial strains, the activity of chitosan and its derivatives were compared with erythromycin as reference antibiotics, while, nystatin was considered the positive control for yeast strain. The inhibition zones of antimicrobial activities were measured as given in Table 3. Although the results showed that *P*. *aeruginosa*, *S*. *aureus* and *B*. *cereus* were resistant to the erythromycin, chitosan and its derivatives presented remarkable activities against these tested bacteria. The results exhibited the significant efficacy of the two Schiff bases against the indicator microorganisms more than the parent chitosan. We could deduce from these findings that Schiff base (I) demonstrated the highest activity in the presence of all examined microorganisms regardless their structures.

**Table 3:** Inhibition indices of chitosan and chitosan Schiff bases (I & II) against *E*. *coli*, *P*. *aeruginosa*, *Salmonella* sp., *S*. *aureus*, *B*. *cereus*, and *C*. *albicans*.

Bacteria	Inhibition zone (mm) in diameter			
	Erythromycin	Chitosan	Schiff base I	Schiff base II
*E*. *coli*	11 ± 0.2	11.5 ± 0.19	17.7 ± 0.16	13.7 ± 0.23
*P*. *aeruginosa*	−ve	12.6 ± 0.13	17.2 ± 0.24	14.4 ± 0.15
*Salmonella* sp.	12.5 ± 0.17	13 ± 0.17	17.1 ± 0.15	14.7 ± 0.14
*S*. *aureus*	−ve	13.9 ± 0.19	18.9 ± 0.21	15.1 ± 0.12
*B*. *cereus*	−ve	13.6 ± 0.16	18.1 ± 0.2	15.9 ± 0.14
**Fungi**	**Nystatin**			
*C*. *albicans*	15.2 ± 0.22	11.4 ± 0.14	15.8 ± 0.25	15.5 ± 0.18

Values are expressed as mean ± SD (n = 3).

The antimicrobial action of chitosan might be differed according to several intrinsic factors including chitosan source, the molecular weight that influences the penetration inside microorganisms, and the synthesis of new chitosan derivatives with novel characteristics that usually improve the antimicrobial action of chitosan. Three principal mechanisms have been posited to elucidate chitosan interaction with various kinds of microorganisms, which vary based on the cell wall structure and metabolic process^23,25^. The first mechanism depends on the chemical interaction via electrostatic manner between the positive charge of amine groups (NH^3+^) of chitosan and the negative charges on the cell wall of various microorganisms, which provokes escape of intracellular ingredients. The second mechanism illustrates the consequence of chitosan molecular weight that controls its penetration into the nuclei of microorganisms and binding with DNA. Therefore, it will result in the suppression of the mRNA expression, which it will consequently prevent the protein synthesis. The third mechanism is ascribed to the chelating capacity of chitosan to metal ions like Ca^2+^, Mg^2+^, and Zn^2+^, which are vital constituents for microbial growth and metabolic pathways such as spore formation in Gram-positive bacteria. Herein, these factors were accomplished in the new chitosan derivatives with medium molecular weight; Accordingly, we propose that the previous mechanisms could be contributed together to implement the antimicrobial potency of the synthesized chitosan derivatives. Earlier studies have reported the development of various Schiff bases utilizing Indole-3-carboxaldehyde to profit its antimicrobial activity, and the newly derivatives showed excellent antibacterial, antifungal, antiparasitic and anticancer activities^50–52^. This illustrates the synergistic boost of chitosan activities in case of Schiff base (I) against the examined microorganisms compared to the synergistic effect of Schiff base (II) to the prime chitosan. As consequence of these findings, MIC, bactericidal and fungicidal activities of the novel chitosan derivatives were subsequently estimated.

### Determination of MIC

**Figure 7:**
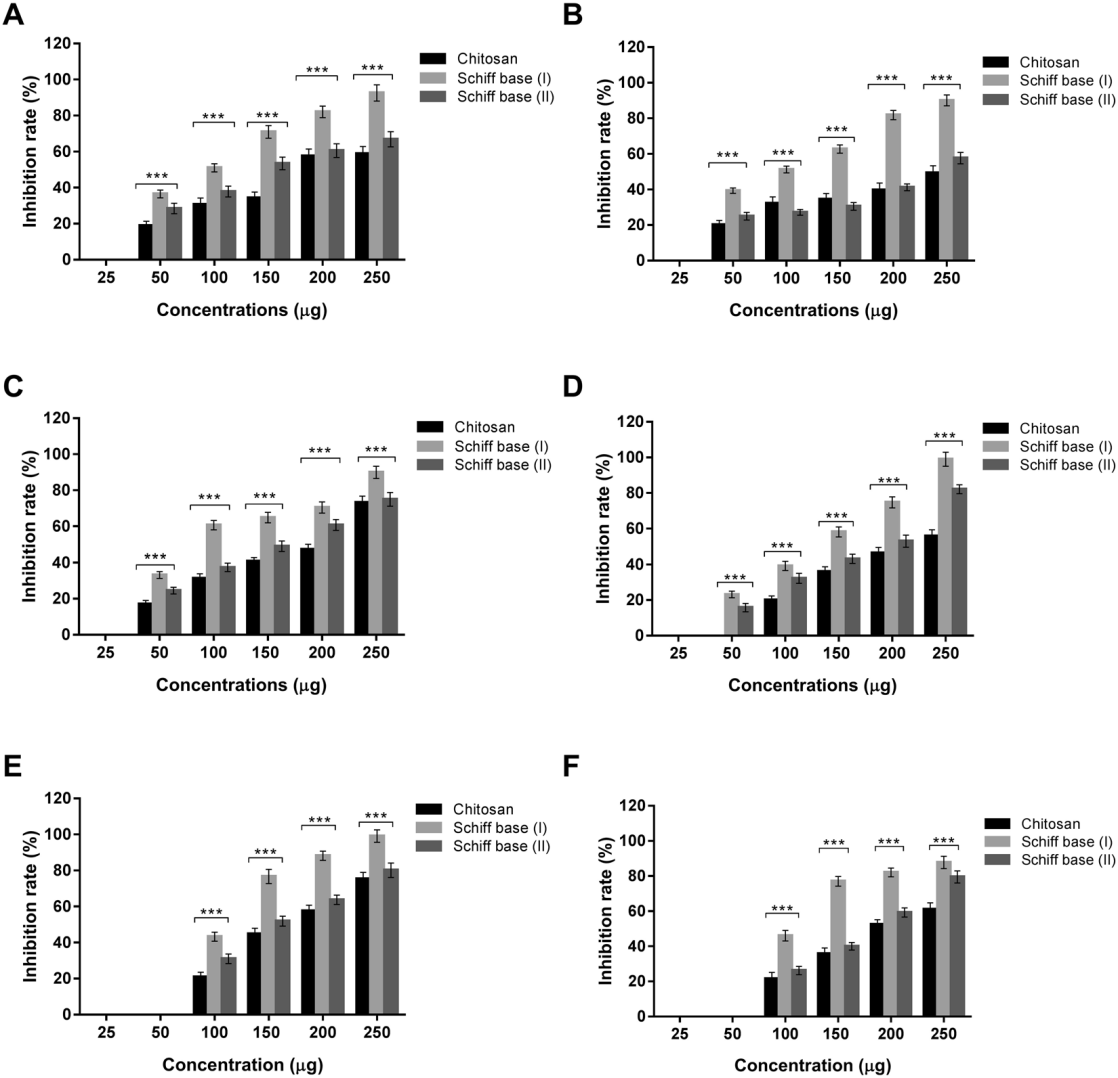
MIC of chitosan and its derivatives Schiff bases (I & II) against **(A)** *E*. *coli*, **(B)** *P*. *aeruginosa*, **(C)** *Salmonella* sp., **(D)** *S*. *aureus*, **(E)** *B*. *cereus* and **(F)** *C*. *albicans*. Values represent means ± SD (***P < 0.05 for chitosan versus Schiff bases (I & II) and Schiff base I versus Schiff base II, n = 6).

In the current research, minimum inhibitory concentrations (MICs) could be defined as the lowest concentrations of chitosan and the developed Schiff bases (I & II) that prevent the growth of the tested strains after overnight incubation. This approach is crucial and targeted to determine the susceptibilities of microorganisms to our studied materials and to evaluate the antimicrobial potency of these new materials. MIC of the two chitosan derivatives were appraised in comparison to native chitosan as presented in Fig. 7A–F. The data indicated that the typical behaviors of microbial growth inhibition were accomplished; where rising levels of the tested polymers increased their antimicrobial activities. It could be deduced from the results that the concentration (25 µg/ml) of all polymers did not show any activity against the microbial cells, while the MIC of chitosan and the new formulations were perceived at 50 µg/ml except in cases of *B*. *cereus* and *C*. *albican* that necessitated 100 µg/ml to impede their proliferation. Moreover, the pure chitosan exerted no inhibition of *S*. *aureus* at 50 µg/ml and required 100 µg/ml as well. Schiff base (I) at the concentration of 50 µg/ml demonstrated the maximum inhibition ratios against *P*. *aeruginosa* and *Salmonella* sp. up to 39.36% and 33.1%, respectively, in comparison to the inhibition percentage of the other microorganisms. As the concentrations of chitosan and its derivatives increased, the inhibition ratios of microbial strains augmented till reaching the maximum with the greatest applied level (250 µg/ml). Accordingly, Schiff base (I) at the concentration of 250 µg/ml illustrated fully inhibition of *S*. *aureus* and *B*. *cereus* with percentage up to 99%, whereas, the maximum inhibition ratio of Gram-negative bacteria reached to 92% in addition to 87.8% in case of *C*. *albicans*. These completely hindrance of microbial growth have not attained using the unmodified chitosan and Schiff base (II). With regard to Schiff base (II), MIC value was observed at concentration of 50 µg/ml for all examined microorganisms excluding *B*. *cereus and C*. *albicans* that required 100 µg/ml for inhibiting their growth. The overall results of Schiff base (II) revealed higher activity than the original chitosan; therefore, 4-dimethylaminobenzaldehyde motivated the antimicrobial activities of chitosan. The overall findings emphasized that the antimicrobial activities of chitosan Schiff bases (I & II) were better than unmodified chitosan against all microorganism that resulted from synergistic effects of chitosan and Schiff bases. Moreover, we derived that Schiff base (I) has the strongest activities against whole indicator microorganisms and this action could be elucidated via boosting the chitosan behavior with Indole-3-carboxaldehyde that clearly showed a synergistic influence on chitosan. This renders Schiff base (I) the most acceptable candidate for further investigations.

Previous study found that MIC values of the pure chitosan were 125 and 500 µg/ml against *S*. *aeureus* and *E*. *coli*, respectively. On the other hand, MIC of the modified chitosan such hydrogel of chitosan and oxalyl bis 4-(2,5-dioxo-2H-pyrrol-1(5H)-yl) benzamide were 125 and 3.91 µg/ml against the same bacterial strains, respectively^32^. Furthermore, another research reported that MIC of O-quaternary ammonium chitosan (OQCS) for *S*. *aureus*, *E*. *coli*, *Aspergillus niger*, *P*. *aeruginosa*, and *Bacillus subtillis* are better than the natiral chitosan in range from 250–600 µg/ml. on the contrary, O-quaternary ammonium N-acyl thiourea chitosan (OQCATUCS) displayed better antibacterial activity than OQCS and chitosan with MIC in range of 125–250 µg/ml^29^. It is worth mentioning that MIC of our developed materials in range of 50–100 µg/ml with strong activity and 250 µg/ml of Schiff base (I) showed complete inhibition rate for Gram-positive and strong inhibition up to 92% for Gram-negative. Our study and the previous reports boost that the modification of pure chitosan with more active groups could promote and extend its antimicrobial activity.

**Figure 8:**
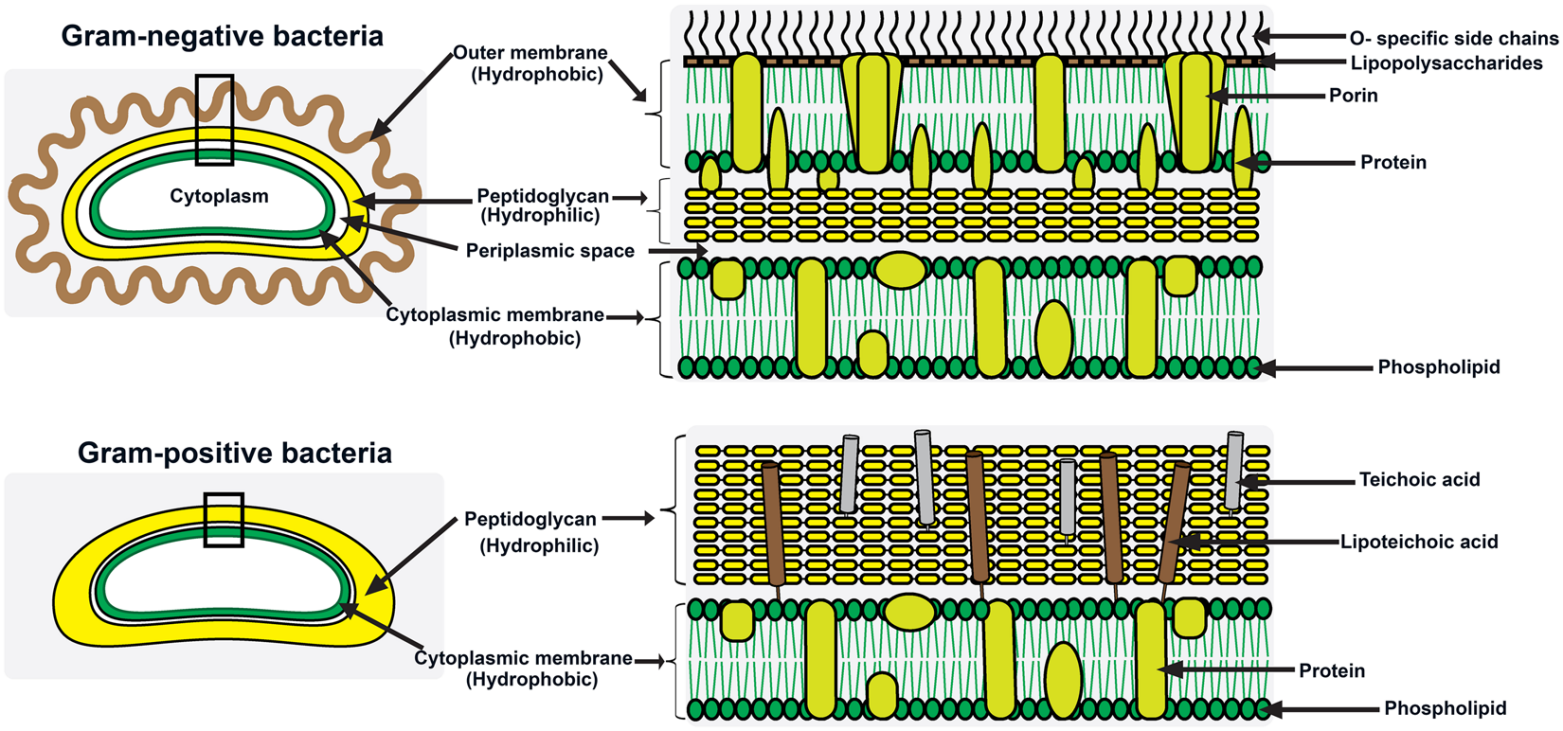
Schematic diagram to illustrate the cell wall structures of Gram-positive and Gram-negative bacteria.

The higher action of Schiff base (I) on Gram-positive bacteria than Gram-negative bacteria that explicitly appeared at 250 µg/ml are due to the difference of the cell wall structures. These behaviours were also observed with the application of Schiff base (II) versus the studied bacterial strains, but with lower activities. These antimcrobial manners are in consistent with previous results^53^. This could be deciphered by two arguemnts; firstly, the presence of three barrier membranes in Gram-negative bacteria that obstruct the penetration of materials iside the cells including hydrophobic outer membrane, peptidoglycan and cell membrane, while the Gram-positive bacteria contain thick peptidoglycan including teichoic acid molecules with negatively charges (Fig. 8).

These strctures of Gram-positive increase their affinity for binding with chitosan that possess positive charges and in consequnce lead to the damage of bacetraial cells. Secondly, the existance of porin channels within the outer membrane of Gram-negative bacteria might hamper the entrance of chitosan residues into the cells. However, the inihibtion ratios of Schiff base (I) at the concentration of 50 µg/ml toward Gram-negative bacteria are greater than Gram-positive bacteria, which can be interpreted as at the high concentration, the most level of derivative attached firmly to the cell wall and the outer surface thtough electrostatic interaction and the excess of polymer enter inside the cells to inhibit the protein synthesis, making it more effective against Gram-positive than Gram-negative. In addition, the well diffusion method corroborates our speculation of this antimicrobial action. Consequently, the antimicrobial activities of the prepared chitosan derivatives could ascribed to the two mechanisms that were mentioned in the previous section depends on the interactions with the cell wall structure and inhibition of protein synthesis via binding with DNA of the cells. For *C*. *albicans*, the cell wall is totally difference consitsing of chitin and glucan. Therefore, it is very stiff and the treatments of fungal strains as all need intensive doses, but the affinity mechanism of chitosan to inhibit the fungal strains is not fully understood compared to the bacterial cells.

### Bactericidal and fungicidal behavior

**Figure 9:**
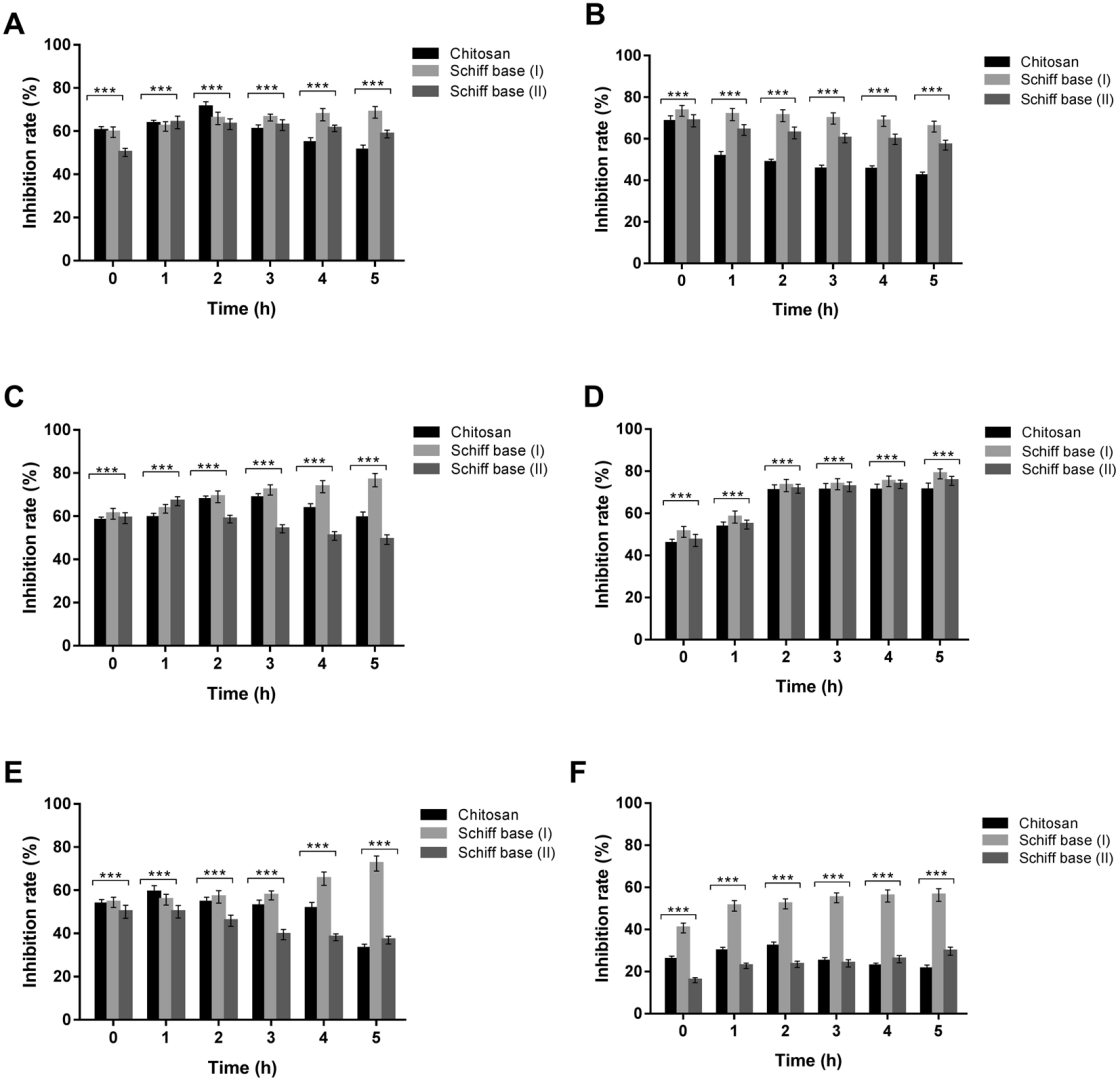
Bactericidal and fungicidal behavior of chitosan and its derivatives Schiff bases (I & II) **(A)** *E*. *coli*, **(B)** *P*. *aeruginosa*, **(C)** *Salmonella* sp., **(D)** *S*. *aureus*, **(E)** *B*. *cereus* and **(F)** *C*. *albicans*.Values represent means ± SD (***P < 0.05 for chitosan versus Schiff bases (I & II) and Schiff base I versus Schiff base II, n = 6).

This study is crucial to identify the manners of microorganisms against new pharmaceutical products to reduce the dose, intervals and duration time. Several factors can affect this investigation such as, material concentration, growth conditions, bacterial density, and duration time. In the present research, 150 µg of native chitosan and the two Schiff bases were evaluated against the pathogenic organisms, which were previusoly mentioned for time intervals. The results of bactericidal and fungicidal assays are illustrated in Fig. 9A–F that show the relations between inhibition rate of microorganisms (%) and the contact time in range of 0–5 h.

From the figures, various performances of bacterial and fungal strains can be observed based on the kind of tested polymers and microorganisms. For Schiff base (I), *P*. *aeruginosa* strated to exhibit a notable resistnace after 1 h, where the inhibtion rate was 73.41% at the first hour and decreased in 5 h to be 65.8%. This action might be due to the high resitsnace of *P*. *aeruginosa* to the most of the famous antibiotics, which renders the treatment process needs a combination of antibiotics to control the growth and hinder the biofilm production that could lead to the cytstic fibrosis and even to the death for the patients. Conversely, the other examined strains revelaed significant senstivity to Schiff base (I) with the increasining of time exposure. In contrast to the results obtained of Schiff base (I), the investigated strains showed resistance to Shciff base (II) that increased with the contact time due to the growing of microorganisms except for *S*. *aureus* and *C*. *albicans* that exhibited augmentations of their susceptibility with the duration time. On the other hand, The pathogenic strains had the ability to grow and divide in the presence of basic chitosan after 5 h in contrast to *S*. *aureus*, where the inhibtion rate of its growth was significantly amplified. It could be elicited from the results that the prepared materials were bacteriostatic and fungistatic toward the studied strains because the inhbition rate within 5 h did not reach to 99.9%. However, Schiff base (I) presneted sigificant activites against the pathogenic strains in comparison to the pure chitosan as a reference material. Therfore, the chitosan derivative (I) should subject to additional investigations including the cellular toxicity assay and biochemical investigations *in vitro* and further *in vivo*.

### Cytotoxicity assay

Cellular toxicity of the prepared materials is one of the crucial characteristics to assess their feasibility for further implementation in biomedical applications through the cell response^54^. Viability assay of the fibroblast cells treated with chitosan and chitosan Schiff bases (I & II) were monitored using MTT assay that has been broadly adopted as a metabolic marker to estimate the cells proliferation compared to the control. Accordingly, the potential toxicity outcomes induced in cells by the examined materials can be determined. In this assay, MTT is reduced by mitochondrial succinate dehydrogenase of viable cells to insoluble formazan crystals, which could solubilize by DMSO to detect the viable cells. Table 4 depicts that the cytotoxicity of chitosan and the two derivatives exerted no significant difference between the effect of chitosan and the Schiff bases in comparison with control cells. The concentration of 100 mg demonstrates approximately 96% of viable cells in the presence of different polymers. Moreover, the highest concentration of the prepared materials reached to 200 mg showed maximum toxicity about 7.8% in the presence of the primitive chitosan. However, the cellular toxicity of Schiff bases (I & II) at 200 mg were 5.2% and 6.3%, respectively. Previous studies reported that the chitosan and the most Schiff bases had inconsiderable harmful to the cells^31,55^. Despite of these toxicities, the cytotoxic values are still in safe levels, which are in correspondence with prior research that proposed 75% of viable cells could be nontoxic materials^15^. The cytotoxicity studies substantiate the safety of the prepared antimicrobial materials and the potentiality of applying them in wound dressing to enhance the wound healing process.

**Table 4:** Cell viability assay of fibroblast cells treated with chitosan and chitosan Schiff bases (I & II).

Polymer concentration (mg)	Viable cells (%)/Chitosan	Viable cells (%)/Schiff base I	Viable cells (%)/Schiff base II
25	99.0 ± 4.2	99.1 ± 3.5	98.8 ± 4
50	97.3 ± 3.7	97.8 ± 3.6	97.1 ± 2.6
100	95.7 ± 2.9	96.4 ± 3	96.1 ± 3.9
150	95.0 ± 3	96.1 ± 4.3	95.7 ± 4.4
200	92.2 ± 2.9	94.8 ± 2.5	93.7 ± 3.1

Values are expressed as mean ± SD (n = 6).

## Conclusion

Chitosan with different function groups (i.e.; hydroxyl and amine groups) has a promising structure to prepare new derivatives. Here, Carbonyl groups of Indole-3-carboxaldehyde or 4-dimethylaminobenzaldehyde was coupled with chitosan amine group to produce Schiff bases (I & II), respectively. The formation of Schiff bases was confirmed by monitoring modifications in the chemical structure using spectroscopic analysis, FT-IR, TGA, DSC. The findings of the antimicrobial activities revealed that antimicrobial activities of chitosan were significantly boosted by Indole-3-carboxaldehyde higher than 4-dimethylaminobenzaldehyde. Furthermore, the cellular toxicity assay pointed to the safety of Schiff bases (I & II). The present findings lead us to further this study via preparing a membrane of Schiff base (I) for investigating *in vivo* as a promising wound dressing candidate to promote the wounds and burns healing process.

## Material and Methods

### Materials

Chitosan (M_W_. 100,000–300,000 Dalton) was obtained from Across Organics. (New Jersey, USA), Indole-3-carboxaldehyde, (purity 97%; M._W_. 145.15) and 4-dimethylaminobenzaldehyde, (purity 99% M._W_. 149.19) were acquired from Sigma Aldrich (Germany). Sodium hydroxide pellets (purity 99–100%), Sulfuric acid (98%), Ethanol (99.9%) were supplied from International co for Supp& Med. Industries, (Egypt).

### Microorganisms

Representative Gram-negative bacteria [*Escherichia coli (E. coli.), Pseudomonas aeruginosa* (*P. aeruginosa*) *and Salmonella sp*.] and Gram-positive [*Staphylococcus aureus* (*S. aureus*), and *Bacillus cereus* (*B. cereus*)] bacteria in addition to *Candida albicans* (*C. albicans*) were utilized to study the antibacterial and antifungal performance of the development materials. All strains were revived from glycerol stocks via growing overnight at 37 °C and 150 rpm in LB broth medium containing (peptone 1%, yeast extract 0.5%, NaCl 1%).

### Preparation of chitosan Schiff base derivatives

The new Schiff base derivatives were synthesized following the authors published work^34^. Firstly, chitosan (1 g) was dissolved in 50 ml of acetic acid (2%) at room temperature with stirring for 6 h. Then, 1.86 mM of aldehyde (Indole-3-carboxaldehyde or 4-dimethylaminobenzaldehyde) was dissolved in 10 ml of ethanol and added dropwise into chitosan solution with continuous stirring at 50 °C for further 6 h. The resultant deep yellow gel was filtered and washed several times with ethanol to remove any un-reacted aldehydes. Finally, neutralization step was achieved using sodium hydroxide (5%) followed by, washing several times with distilled water. The obtained Schiff bases were dried overnight at 60 °C in a vacuum oven.

### Physicochemical characterization

The changes in the chemical structures and surface morphologies of the prepared chitosan Schiff bases were investigated by Fourier Transform Infrared Spectrophotometer (FT-IR; Model 8400 S, Shimadzu, Japan) and Scanning Electron Microscope (SEM; Model Jsm 6360 LA, Joel, Japan). While, their thermal properties were examined using Thermal Gravimetric Analyzer (TGA; Model 50/50H, Shimadzu, Japan) and Differential Scanning Calorimetric (DSC; Model 60A, Shimadzu, Japan). Moreover, the electronic spectra of the developed chitosan Schiff bases were identified via UV-Vis Spectrophotometer (Ultrospec 2000 Pharmacia BiotechCo., Cambridge, England) in scanning ranged using 0.2% chitosan or its new Schiff base derivatives. The electronic absorbance was investigated in the scanning range 200–500 nm^43^.

On the other hand, the free amine content was estimated using potentiometric titration, where samples (0.1 g) were dissolved in 20 ml of HCl (0.1 N) and kept overnight (12 h) under stirring at room temperature to provide a sufficient time for polymer hydration. Thereafter, the solution was titrated with NaOH (0.1 N). The degree of deacetylation was calculated using Eq. (1) and (2)^56^.

1 $$NH2=\frac{203.195\times {\rm{w}}(NH2)}{16.0262+0.42037\times w(NH2)}$$

2 $${\rm{w}}(NH2)=\frac{V\times c\times 100\times 0.016}{Wdry}$$

where V is the consumed volume of NaOH between two abrupt changes of pH, C is the concentration of the used NaOH and W_dry_ is the dry weight sample. While, NH_2_ and W_(NH2)_ were estimated as the percentage values.

### Antimicrobial assay using Agar-well diffusion method

Agar-well diffusion approach was adopted to assess the antimicrobial activities of pure chitosan, and chitosan derivatives against various pathogenic microorganisms (*E*. *coli*, *P*. *aeruginosa*, *Salmonella* sp., *S*. *aureus*, *B*. *cereus* and *C*. *albicans*) as previously described^57^. Old overnight cultures of the tested microorganisms were diluted 10-fold in LB broth free medium and their turbidities were adjusted to be equal the McFarland 0.5 standard via measuring at 625 nm, where bacterial strains were 1–2 × 10^8^ CFU/ml, while *C*. *albicans* was 1–5 × 10^8^ CFU/ml. Then, 50 µl of the cell suspensions were spread over the surface of LB agar plates using glass spreader. The Agar plates were bored with a metal cork borer to produce wells of 6 mm in diameter, and 50 µl of the examined biopolymers were loaded. Afterwards, the Petri dishes were kept in the fridge for 2 h to diffuse the materials into the agar. The plates were aerobically maintained at 37 °C for 18 h, and the antimicrobial activities were then determined via estimating the inhibition zone of microbial growth. Our previous report proved the efficiency of chitosan to inhibit the growth of several microorganisms; therefore, it considers positive control. Moreover, reference antibiotics including erythromycin (15 µg/ml) and nystatin (100 units) discs were applied for bacteria and *Candida* experiments, respectively. This assay was adopted as a screening approach to determine whether the prepared Schiff bases have siginficant inhibition against the indicator microorganisms to apply the further studies or the further should be neglected. Therefore, this antimicrobial evaluation for the investigated biopolymer was implemented in triplicate.

### Determination of minimum inhibitory concentration (MIC)

Microtiter plate method is one of the most effective approaches for determining the MIC for various antimicrobial agents. Consequently, it was conducted to investigate the impact of different concentrations of chitosan and the new derivatives on the growth of the indicator microorganisms as previously demonstrated^58,59^.

The tested biopolymers were sterilized by 0.22 µm syringe filter previous to use. The overnight bacterial cultures were diluted 100-folds in LB broth free medium to optical densities of 0.9 for all microorganisms via measuring the bacterial turbidity at 600 nm. Afterward, 20 µl of the bacterial culture suspensions were inoculated into a sterile 96-well microplate, and various levels of filtered native chitosan and modified chitosan (25, 50, 100, 150, 200 and 250 µg/ml) were incorporated. The wells were then completed with LB broth free up to 200 µl followed by mixing well employing a bench shaker for 2 min at 100 rpm and incubated aerobically at 37 °C for 24 h. The positive and negative controls were set by combining the tested materials only and the diluted bacterial suspension only with free LB, respectively. The microtiter plates were agitated for 30 seconds using a microplate reader, and the absorbance was measured at 600 nm to determine the turbidity of the different bacterial cultures. The antimicrobial assays were executed in six replicates. The mean and standard deviations (SD) were calculated. The percentages of microbial growth inhibition were estimated using Eq. (3).

3 $${\bf{G}}{\bf{r}}{\bf{o}}{\bf{w}}{\bf{t}}{\bf{h}}\,{\bf{I}}{\bf{n}}{\bf{h}}{\bf{i}}{\bf{b}}{\bf{i}}{\bf{t}}{\bf{i}}{\bf{o}}{\bf{n}}\,{\bf{r}}{\bf{a}}{\bf{t}}{\bf{i}}{\bf{o}}\,( \% )=\frac{({\rm{OD}}\,{\rm{of}}\,{\rm{normal}}\,{\rm{microbial}}\,{\rm{growth}}-{\rm{OD}}\,{\rm{of}}\,{\rm{inhibited}}\,{\rm{microbial}}\,{\rm{growth}})}{({\rm{OD}}\,{\rm{of}}\,{\rm{normal}}\,{\rm{microbial}}\,{\rm{growth}})}\times 100$$

### Study of Bactericidal and fungicidal performance

To evaluate the bactericidal and fungicidal behaviour of chitosan and its derivatives, microtiter plate approach was conducted by adapting the previous procedure^60^. The overnight cultures of the indicator microorganisms were diluted with LB medium as mentioned above to obtain optical densities of 1.2 at 600 nm for various cultures. One ml of each bacterial suspension was thoroughly mixed with 150 µg of chitosan and its derivatives. The mixtures were incubated at 37 °C for different times (0, 1, 2, 3, 4 and 5 h), and then, 10 µl from each tube was inoculated in 96-wells microplates. Subsequently, the wells were filled with LB broth up to 200 µl. The microplates were quite shaken and incubated at 37 °C for 24 h. After incubation, the turbidities of the bacterial cultures were measured at 600 nm using microplate reader as described above. All measurements were performed in six determinations, and the mean and SD were estimated.

### Cytotoxicity assay *in vitro*

The cell viability test was performed on the mouse fibroblast cell line (NIH 3T3) and estimated using MTT [3-(4,5-Dimethythiazol-2-yl)-2,5-Diphenyltetrazolium Bromide] assay^61,62^. The fibroblast cells were grown in 25 cm^2^ culture flask including complete Dulbecco’s modified Eagle’s medium (DMEM) supplemented with 10% fetal bovine serum. The cells were fostered in a CO_2_ incubator at 5% CO_2_ and 37 °C with 85% humidity. When the cell confluency reached 80%, the cells were washed with PBS and harvested by trypsinization method using 0.5% trypsin. Afterwards, The cells were counted utilizing trypan blue by haemocytometer under a light microscope to differentiate between the dead and viable cells. The fibroblast cells were treated with different concentrations (25, 50, 100, 150, and 200 mg/200 µl of media) of chitosan and chitosan Schiff bases (I & II) Powder. Chitosan and its Schiff bases derivatives were sterilized via embedding in 70% ethanol and allowed to dried under UV for 30 min^63^. Then, the biopolymer samples were washed several times with PBS before applying to cells. The fibroblast cells were cultured in a 96-well tissue culture plate at 3 × 10^3^ cells/well. The total volume of each well including the tested polymer was 200 µl, whereas, the control wells containing the fibroblast cells without treatment. The cytotoxicity examination was performed in triplicate for each sample, and the plate was incubated in the CO_2_ incubator under the previous conditions for 2 days. After that, the medium was removed, and the wells were washed twice with PBS to remove the materials and cell debris. Subsequently, 20 µl of MTT (5 mg/ml) was added to each well, and the plate was agitated for 5 min at 120 rpm for mixing thoroughly. The plate was further incubated at 37 °C for 4 h in CO_2_ incubator to convert the MTT to formazan. The supernatant was then discarded, and 200 µl of dimethylsulfoxide (DMSO) was then added to each well for dissolving the formazan crystals. The plate was agitated for 5 min at 120 rpm, and the absorbance was gauged at 570 nm using microtiter plate reader to estimate the percentage of viable cells. The results were recorded, and the percentage of cell viability of the treated cells with examined materials was estimated by comparing with the untreated cells as a control using Eq. (4).

4 $${\bf{C}}{\bf{e}}{\bf{l}}{\bf{l}}\,{\bf{v}}{\bf{i}}{\bf{a}}{\bf{b}}{\bf{i}}{\bf{l}}{\bf{i}}{\bf{t}}{\bf{y}}\,{\bf{r}}{\bf{a}}{\bf{t}}{\bf{i}}{\bf{o}}\,( \% )=\frac{{\rm{As}}}{{\rm{Ac}}}\times 100$$

where (As) is the absorbance of treated cells with of tested material, while (As) is the absorbance of untreated cells. The measurements were carried out in six replicates.

### Statistical analysis

All experiments were carried out in triplicate, and the results were statistically analyzed employing GraphPad Prism software (Version 5). The data were analyzed using two-way analysis of variance (ANOVA) with Tukey test for multiple comparisons. The values represent the means ± SD of each group. Multiple comparisons were done for chitosan versus Schiff bases (I & II) and Schiff base (I) versus Schiff base (II). The significant data were determined at P-value < 0.05 (n = 6).
